# Fabrication of High-Performance Thin-Film Composite Nanofiltration Membrane by Dynamic Calcium-Carboxyl Intra-Bridging during Post-Treatment

**DOI:** 10.3390/membranes10070137

**Published:** 2020-06-30

**Authors:** Hongyi Han, Ruobin Dai, Zhiwei Wang

**Affiliations:** State Key Laboratory of Pollution Control and Resource Reuse, Shanghai Institute of Pollution Control and Ecological Security, School of Environmental Science and Engineering, Tongji University, Shanghai 200092, China; hanhy1219@163.com (H.H.); dairuobin@163.com (R.D.)

**Keywords:** post-treatment, nanofiltration, calcium-carboxyl intra-bridging, water purification, desalination

## Abstract

Widespread applications of nanofiltration (NF) and reverse osmosis (RO)-based processes for water purification and desalination call for high-performance thin-film composite (TFC) membranes. In this work, a novel and facile modification method was proposed to fabricate high-performance thin-film composite nanofiltration membrane by introducing Ca^2+^ in the heat post-treatment. The introduction of Ca^2+^ induced in situ Ca^2+^-carboxyl intra-bridging, leading to the embedment of Ca^2+^ in the polyamide (PA) layer. This post modification enhanced the hydrophilicity and surface charge of NF membranes compared to the pristine membrane. More interestingly, the modified membrane had more nodules and exhibited rougher morphology. Such changes brought by the addition of Ca^2+^ enabled the significant increase of water permeability (increasing from 17.9 L·m^−2^·h^−1^·bar^−1^ to 29.8 L·m^−2^·h^−1^·bar^−1^) while maintaining a high selectivity (Na_2_SO_4_ rejection rate of 98.0%). Furthermore, the intra-bridging between calcium and carboxyl imparted the NF membranes with evident antifouling properties, exhibiting milder permeability decline of 4.2% (compared to 16.7% of NF-control) during filtration of sodium alginate solution. The results highlight the potential of using Ca^2+^-carboxyl intra-bridging post-treatment to fabricate high-performance TFC membranes for water purification and desalination.

## 1. Introduction

Water resources are distributed unevenly worldwide. As the rapidly increasing population and water consumption in many areas of the world, nearly one third of world’s population live under water scarcity. Water purification and desalination can be an effective route to address the water shortage and crisis worldwide [1,2,3,4]. Membrane-based processes have attracted much attention due to their high efficiency for wastewater reclamation, desalination, and water purification [5,6]. Nanofiltration (NF) and reverse osmosis (RO)-based processes play a dominant role in water harvesting applications [7,8,9]. In these applications, thin-film composite (TFC) membranes have experienced the tremendous development for a few decades and each layer of TFC membrane can be independently controlled and optimized to achieve expected selectivity and permeability. The membranes hold the key to the performance and cost-effectiveness of the processes [10,11].

The structure of the commercial TFC polyamide (PA) membrane is typically composed of three layers [12], i.e., the bottom layer (nonwoven fabric) serving as mechanical support, the middle layer usually prepared by polyethersulfone (PES) or polysulfone (PSF) acting as porous substrate for interfacial polymerization (IP) and the top PA selective layer about 10–200 nm thickness formed via IP process [13]. The IP process refers to a polymerization reaction that occurs at the interface of two different monomers dissolved in two immiscible solutions. The PA layer largely determines the permeability and selectivity, and therefore intensive efforts have been dedicated to regulating the IP process for fabricating a high-performance TFC membrane [14,15,16]. 

In a practical fabrication process, an immediate post-treatment was further applied after the IP reaction [17]. It has been reported that the post-treatment process induces further impacts on the structure and performance of TFC PA membranes. In general, the post-treatment can dynamically promote the cross-linking process between monomers, shrink pores of the substrate, increase the growth rate of polymer chains, and stabilize the structure of PA layer [18,19]. For instance, Maria et al. fabricated TFC PA membranes via IP followed by solvent activation, which resulted in the increase of flux [20]. Han et al. used different temperature and time in the post-treatment to improve rejection of NF membranes [21]. 

Based on the dynamic nature of the post-treatment process, we hypothesize that membrane modification could be incorporated directly into the post-treatment, thus enabling in situ modification for improving the membrane separation performance. There have been some studies relevant to introducing various ions into IP, for example, Hao et al. reported a fouling mitigation approach for forward osmosis (FO) and RO membranes via Ca^2+^ added during the IP process [22,23]. However, currently the ions have not been introduced into the post-treatment process. 

Inspired by this, we proposed to use the intra-bridging between calcium and carboxyl groups in PA layer for modification of NF membrane during post-treatment, since calcium ion is capable of complexing with carboxyl groups [23]. During the post-treatment process, the intra-bridging might result in the embedment of Ca^2+^ into the PA layer, induce the change of physicochemical properties of membranes and thus enhance the separation performance. In this work, surface morphology, chemical composition, and separation properties of the modified membrane were systematically investigated, and the mechanisms in enhancing permeability and antifouling property were elucidated. The dynamic modification method in our work paves a new route to fabricate high-performance TFC membranes for water purification and desalination. 

## 2. Materials and Methods 

### 2.1. Materials

PES membrane (LX-300K, MWCO = 300 kDa), which was used as the substrate for forming PA layer, was provided by Synder Filtration. Piperazine (PIP, 99%), 1,3,5-benzenetricarbonyl trimesoyl chloride (TMC, 98%), and *n*-hexane used for IP process were purchased from Aladdin (Shanghai, China). Calcium chloride (CaCl_2_, AR) from Macklin (Shanghai, China) was used in the post-treatment. Sodium sulfate (Na_2_SO_4_, AR) was used as the salt solute for NF tests. Sodium alginate (SA, AR) was adopted as organic model foulants for antifouling tests.

### 2.2. Membrane Fabrication 

**Figure 1:**
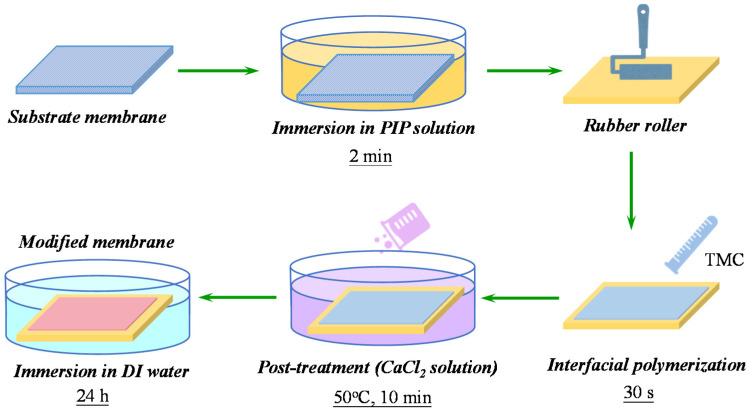
Schematic of the fabrication process of thin-film composite nanofiltration (NF) membrane via dynamic calcium-carboxyl intra-bridging during post-treatment.

The NF membrane was prepared by a typical IP process followed by a dynamic calcium-carboxyl intra-bridging during post-treatment, which is shown in Figure 1. Prior to IP, the porous PES substrate was soaked into deionized water for at least 12 h before use. The PES substrate was removed from deionized water and dried by Kimberly tissue. Afterwards, the substrate was soaked in aqueous solution of PIP (1.0 wt%). After 2 min immersion, the residual PIP solution on the substrate was squeezed by a rubber roller. Then the *n*-hexane solution containing TMC (0.15 wt%) was poured onto the surface of PES substrate. After reaction for 30 s, the TMC/n-hexane solution was poured out and excess solution removed upon volatilization. Then the as-formed TFC PA membrane was transferred to 50 °C water bath which contains different concentration of CaCl_2_ (0, 10, 20, 40, and 80 g/100 mL). The membrane was cured in the water bath for 10 min. Finally, the prepared membranes were thoroughly washed by DI water to remove any residual CaCl_2_ and further stored in DI water at 4 °C before characterization and performance test. The resulting membranes were denoted as NF-control, NF-10, NF-20, NF-40, NF-80, respectively, based on the concentration of CaCl_2_ used in post-treatment. 

### 2.3. Membrane Characterization

The surface morphology of the composite nanofiltration membranes was observed by scanning electron microscope (SEM, Hitachi S-4800, Minato-Ku, Japan) with an acceleration voltage of 5.0 kV and platinum was sputtered on the surface to achieve the minimum conductivity for a valid SEM observation. An atomic force microscope (AFM, Veeco NanoScope MultiMode III, Santabarbara, CA, USA) was used to detect the surface roughness of the polyamide selective layer in the peak force trapping mode. The surface elemental composition of the NF membrane was detected by X-ray photoelectron spectroscopy (XPS, PHI 5000C ESCA System, Lafayette, LA, USA) with the calibration using C1s = 284.6 eV as a reference [24]. Attenuated total reflectance Fourier transform infrared spectroscopy (ATR-FTIR, Nicolet 6700, Thermo Fisher Scientific Inc., Waltham, MA, USA) was used to analyze the chemical structures of membranes. The water contact angle of NF membranes was determined by a sessile drop method (OCA 15 Plus, Data Physics GmbH, Filderstadt, Germany). Zeta potential of all membranes was detected using a 1 mM KCl solution at pH = 7 and 10 by a potentiometric analyzer (SurPASS 3, Anton Paar, Ashland, Viginia, USA). The salt concentrations of both the feed solution and permeate solution in NF performance test were determined by a conductivity meter (DDSJ-308F, INESA instrument, Shanghai, China). Membrane fouling was tested using sodium alginate (SA) as model organic foulants to represent polysaccharides. The applied pressure was adjusted to maintain an initial flux of 228 L·m^−2^·h^−1^ for all fouling experiments. 

### 2.4. Nanofiltration Performance Tests 

The NF performance of resulting membranes was characterized by measuring the pure water flux and salt rejection. Experiments were carried out in a cross-flow filtration cell with effective area of 6.3 cm^2^ [24]. All NF performance tests were performed for three times. The concentration of Na_2_SO_4_ solution, which was used to test the rejection rate, was 10 mmol/L. Each membrane was initially pre-compacted at 10 bar for 4 h, and then the pressure was adjusted to the operating pressure of 8 bar to determine the NF performance. The water permeability (PWP) and salt rejection (*R*) were determined by Equations (1) and (2), respectively.

(1) $$\mathrm{PWP}=\frac{\Delta V}{A\times t\times P}$$

where ∆*V* (L) is the volume of permeate solution, *A* (m^2^) is the effective area of the PA NF membrane, t (h) is the testing time, and *P* is the operating pressure (bar).

(2) $$R=(1-\frac{C_{p}}{C_{f}})\times 100\%$$

where *C*_f_ (mg/L) and *C*_p_ (mg/L) refer to the Na_2_SO_4_ concentrations of the feed and permeate solutions, respectively. 

## 3. Results and Discussion 

### 3.1. Membrane Surface Morphology

**Figure 2:**
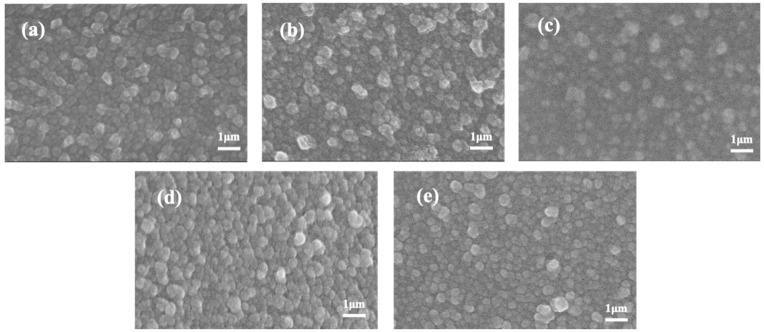
SEM characterization of thin-film composite NF membranes with different concentrations CaCl_2_ added in the post-treatment: (**a**) NF-control, (**b**) NF-10, (**c**) NF-20, (**d**) NF-40, (**e**) NF-80.

Various NF membranes were synthesized via an IP process followed by the dynamic thermal post-treatment with addition of CaCl_2_. The surface morphology and roughness of the resulting membranes were characterized by SEM and AFM, respectively. The surface morphology of NF membranes with different concentrations of CaCl_2_ added in water bath during post-treatment is shown in Figure 2. The images demonstrate that the PA selective layers had a nodular structure, which is the typical structure of PIP based PA layer [25,26]. Compared with the control NF membrane, the surface morphology of membranes upon dynamic post-treatment changed obviously with adding Ca^2+^. It seems that the surface of NF-40 had the most significant nodular-structured morphology. With the increase of CaCl_2_ concentration used for dynamic post-treatment, Ca^2+^ in the surface of membrane would saturate as shown in the results of XPS. Therefore, the NF-40 possessed the most significant nodular-structured morphology, while the nodular structure of NF-80 was not obviously changed compared to those of NF-control and NF-10. After IP process of the NF membrane fabrication, there were some residual solutions of PIP/water and TMC/*n*-hexane during post-treatment. A possible intra-bridging [27] between calcium ions and carboxyl groups in PA matrix might account for the change in membrane surface morphologies. Moreover, heat-treatment could induce further cross-linking for membranes and removal of residual organic solvent [19,28]. The above-mentioned reasons explained why the structure of the modified membrane was different from the nascent membrane.

**Figure 3:**
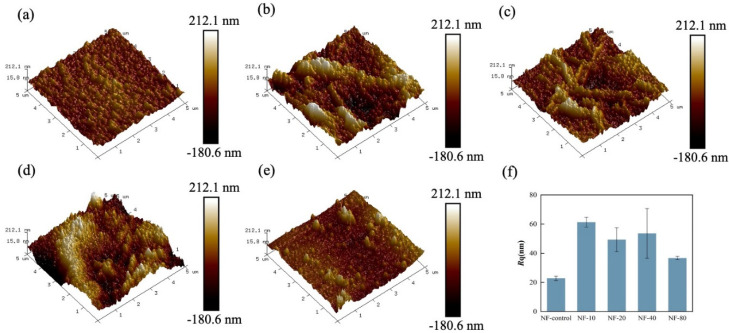
AFM micrographs of all resulting membranes: (**a**) NF-control, (**b**) NF-10, (**c**) NF-20, (**d**) NF-40, (**e**) NF-80; (**f**) the roughness of fabricated membranes.

The AFM images revealing the surface morphology and the average surface roughness (*R*_q_). are shown in Figure 3. The NF-control membranes had the smoothest surface with *R*_q_ = 22.8 nm. In contrast, the roughness of modified membranes sharply increased when calcium ions were added in the water bath. In post-treatment, with the further polycondensation reaction between two monomers, the surface morphology of the PA selective layer became rougher because of the formation of nodular structure promoted by calcium ions [18]. Residual solution continued to react during post-treatment and thus changed the degassing behavior which affected the morphology of the modified membrane [29]. However, the roughness of NF-80 decreased, which is consistent with the results of SEM (Figure 2) due to the decrease of the available calcium (the results of XPS). Furthermore, the complexation of calcium ions with the carboxyl groups of PA layer resulted in an unevenly distributed nodular morphology, contributing to the increase of surface roughness. 

### 3.2. Chemical Composition of Polyamide Layer 

**Figure 4:**
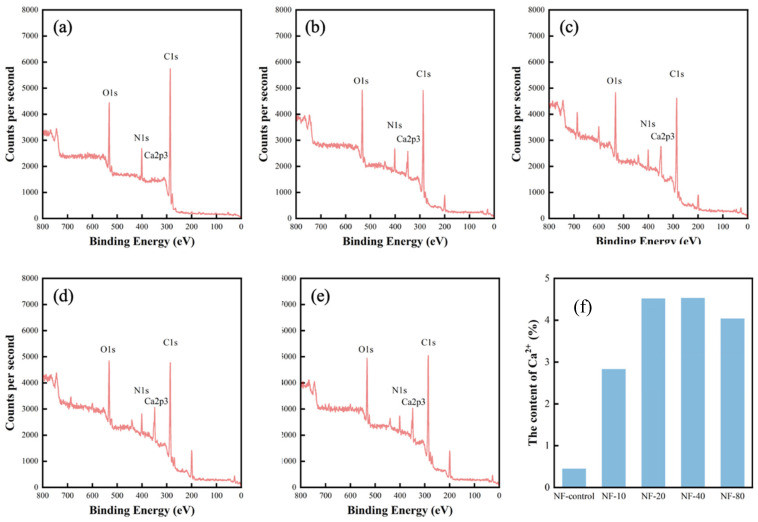
XPS spectra of the thin-film composite nanofiltration with different concentrations CaCl_2_ added in the post-treatment: (**a**) NF-control, (**b**) NF-10, (**c**) NF-20, (**d**) NF-40, (**e**) NF-80; (**f**) the element of Ca^2+^ for different membranes.

The element compositions (including carbon, nitrogen, oxygen, and calcium) of PA selective layer were detected by XPS. As shown in Figure 4, the main element compositions on the membrane surface were C1s, N1s, O1s, and Ca2p3 with peaks centered around 284, 399, 532, and 340 eV. It indicated that Ca content of membranes increased with the increase of CaCl_2_ concentration in the dynamic modification. The intensity of Ca2p3 peak reached the highest value (4.53%) for NF-40 membrane and then decreased slightly as shown in Table 1. This result suggests that the PA layer of resulting membrane had an saturated Ca^2+^ embedment due to the certain amount of residual carboxyl groups [30,31]. 

**Figure 5:**
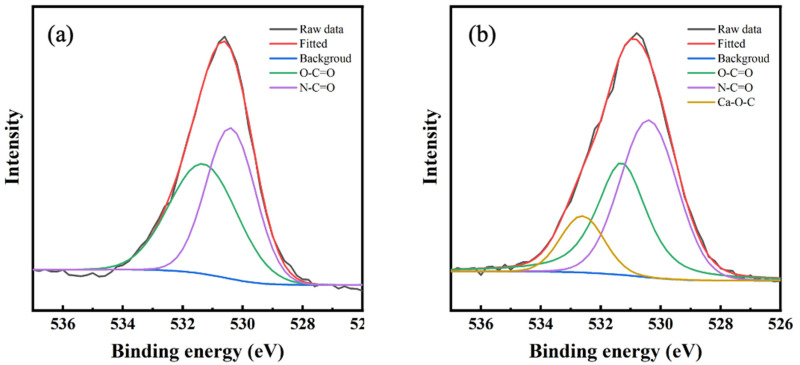
XPS spectra of oxygen 1s in high-resolution spectra: (**a**) NF-control; (**b**) NF-40.

High-resolution oxygen (1s) XPS spectra of NF-control and NF-40 membranes was deconvoluted to further analyze chemical bonding of the PA layer. It showed that there were two peaks at 530.4 eV and 531.3 eV in Figure 5a, suggesting the presence of two types of oxygen in the PA layer of NF-control membrane [31]. The former peak represents carboxylic oxygen groups (O*-C=O) and the latter is ascribed to amide oxygen groups (N-C=O*). Compared with NF-control, another peak appeared at 532.6 eV in Figure 5b, which is associated with calcium species containing coordination bond (C-O*-Ca). The results provided strong evidence that the Ca^2+^ had been successfully incorporated into the selective layer. Furthermore, the ratio of O*-C=O of NF-40 membrane decreased in comparison to that of NF-control membrane, due to possible competitive effect of Ca^2+^ bonding with carboxyl groups. Therefore, it can be inferred that Ca^2+^ was chemically bonded in the PA layer [30]. 

**Figure 6:**
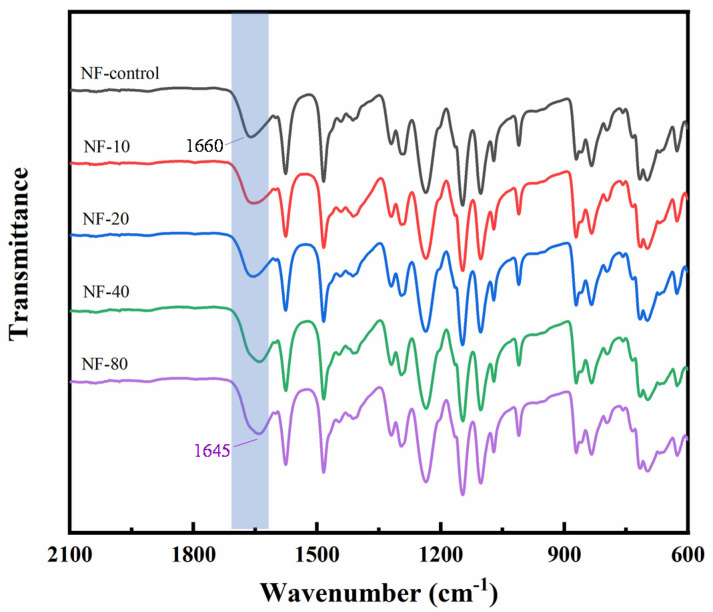
ATR-FTIR spectra of the thin-film composite NF with different concentrations CaCl_2_ added in the post-treatment.

In order to further confirm the complexation of Ca^2+^ with the carboxyl groups in PA selective layer, the FTIR spectra for resulting membranes were measured (Figure 6). Generally, the band of bending vibration of N-H (amide II peak) is located at 1576 cm^−1^, while the characteristic peak at 1660 cm^−1^ is assigned to the stretching vibration of C=O (amide I peak), indicating the presence of functional groups of PA selective layer [32,33]. The spectra showed that the peak of C=O had a shift from 1660 cm^−1^ to 1655 cm^−1^, 1648 cm^−1^, 1645 cm^−1^, and 1645 cm^−1^ with the increase of Ca^2+^ concentrations, respectively. However, the characteristic peak of N-H had no shift in all groups. It implied that the shift of characteristic peak of C=O should be related to the complexation between Ca^2+^ and the carboxyl groups of PA selective layer. CaCl_2_ had a stronger electron-withdrawing effect on C=O than hydrogen bonding of N-H, which led to a shift of C=O band to a lower frequency [34,35]. In combination with the results of XPS, it further demonstrated that when the concentration of Ca^2+^ increased to an extent (NF-40 in this study), the complexation between Ca^2+^ and carboxyl groups reached saturation and further increase of CaCl_2_ concentration did not lead to the embedding of more Ca^2+^ in the PA selective layer. 

### 3.3. Surface Charge and Hydrophilicity 

**Figure 7:**
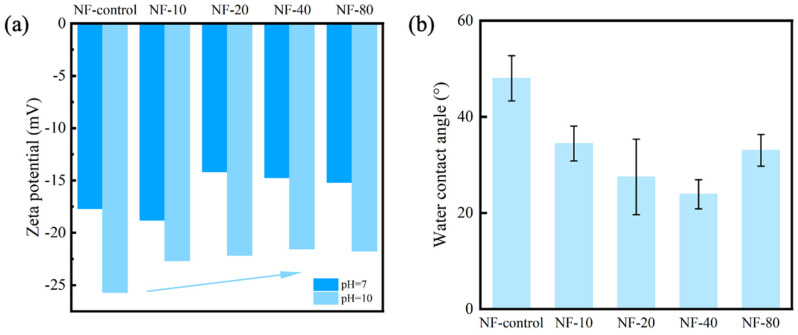
(**a**) Zeta potential of NF-control, NF-10, NF-20, NF-40, and NF-80; (**b**) Water contact of NF-control, NF-10, NF-20, NF-40, and NF-80.

The surface charge property of NF membrane is an important factor affecting rejection rate of charged solutes. Figure 7a shows the zeta potential of NF-control, NF-10, NF-20, NF-40, and NF-80 at different pH values (pH = 7 and pH = 10). In general, it shows that the PA selective layer was less negatively charged with the increase of concentration of CaCl_2_. NF-control had the most negative zeta potential about −25.7 mV (pH = 10), which was due to the deprotonated carboxyl group presenting negative charge (i.e., deprotonation of carboxylic acid groups) on the PA layer. NF-40 showed the least negative zeta potential around −21.5 mV (pH = 10), attributed to the partial charge screening effect upon the complexation between Ca^2+^ and carboxyl groups. At pH = 7, NF-10 had the most negative zeta potential. Statistical analysis by SPSS showed that there was no significant difference of NF-control and NF-10, which indicated that the results of zeta potential at pH = 7 and 10 were generally consistent.

The hydrophilicity of NF membrane was characterized by water contact angles via a sessile drop method. As shown in Figure 7b, the water contact angles dropped sharply and then increased. A hydrophilic surface can grant the membrane with antifouling performance [36,37]. The NF-control membrane had the largest water contact angle of 48.0° ± 4.7° and the lowest roughness. For comparison, the water contact angle of NF-40 was the lowest (23.9° ± 3.0°), indicative of the highest hydrophilicity. The dramatic changes in hydrophilicity was ascribed to a significant change of the physicochemical environment with Ca^2+^ addition (e.g., changing hydrogen bonding behavior and accommodating hydration water molecules by Ca^2+^) [23]. 

### 3.4. Mechanisms of Dynamic Modification Method

**Figure 8:**
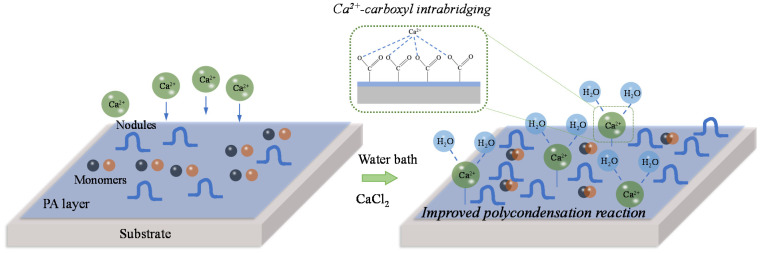
Schematic of mechanisms for Ca^2+^-carboxyl intra-bridging and modification during post-treatment.

The possible mechanisms of dynamic modification are shown in Figure 8. Two main processes occur simultaneously in the post-treatment process based on Ca^2+^-carboxyl intra-bridging involving the formation of Ca^2+^-carboxyl chemical bonds and regulation of cross-linking by the presence of Ca^2+^. In the presence of Ca^2+^, the positively charged Ca^2+^ could easily complex with the negatively charged carboxyl groups in the PA matrix due to initial electrostatic interaction and further formation of coordination bonds (in the four-coordination or six-coordination) in the PA layer, which screened the negative surface charge of NF membrane [23]. Moreover, further polycondensation reaction between residual PIP and TMC would occur due to the increase of temperature, which could thermodynamically promote the reaction forward. It facilitated the formation of more nodules on the surface and incorporating of Ca^2+^ into PA matrix surrounded (or intra-bridged) by carboxyl groups. The intra-bridging made the surface of NF membrane more hydrophilic. Ca^2+^ may also interact with H_2_O leading to the hydration of membrane, which can also improve the hydrophilicity. Furthermore, the complexation between Ca^2+^ and carboxyl groups avoided the exposure of carboxyl groups on the PA matrix, contributing the mitigation of membrane fouling. 

### 3.5. Separation Performance of the Composite NF Membranes 

**Figure 9:**
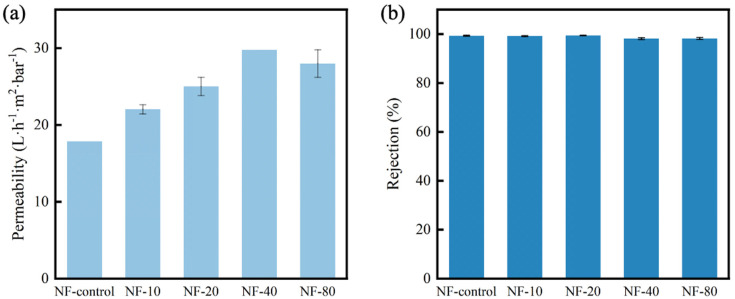
The separation properties of membranes with different concentrations CaCl_2_ added in the post-treatment: (**a**) water permeability, (**b**) Na_2_SO_4_ rejection.

Separation performance, including water permeability and Na_2_SO_4_ rejection, were measured by crossflow filtration at the pressure of 8 bar, with the results shown in Figure 9. The NF-control membrane had the lowest permeability of 17.86 L·m^−2^·h^−1^·bar^−1^. In comparison, the membrane of NF-40 showed the highest permeability of 29.76 L·m^−2^·h^−1^·bar^−1^, which increased by 67% compared with that of NF-control membrane. The change in water permeability could be ascribed to the improved hydrophilicity and increased nodular structure of the modified PA selective layer. 

Inorganic salt Na_2_SO_4_ was applied to assess the solute rejection of the membrane. With the increase of water permeability, all the membranes still maintained almost the same salt rejection around 98%. Mechanisms governing the salt rejection of nanofiltration membrane typically include Donnan exclusion and steric hindrance [35]. As abovementioned (Figure 7), the zeta potentials showed that the surface charges of calcium contained membranes were higher than that of NF-control membrane, indicating a possible decreased electrostatic repulsion. Therefore, it can be inferred that intra-bridging between CaCl_2_ and carboxyl groups can narrow the pore size of NF membranes (which was evidenced by denser morphology of CaCl_2_ added membranes) with increased steric hindrance, which thus maintained the salt rejection rates. Note that the water permeability of calcium contained membranes still increased when the steric hindrance increased, highlighting the positive role of the improved hydrophilicity and nodular structure induced by Ca^2+^-carboxyl intra-bridging.

We further compared the performance of NF-40 with NF membranes in literature with different conditions of post-treatment in terms of water permeability and Na_2_SO_4_ rejection, with the results listed in Table 2. The NF-40 membrane demonstrated better performance compared to those in literature, suggesting that dynamic modification using Ca^2+^ had great potential as a feasible post-treatment method to fabricate NF membranes with both high permeability and selectivity. 

### 3.6. Antifouling Performance

**Figure 10:**
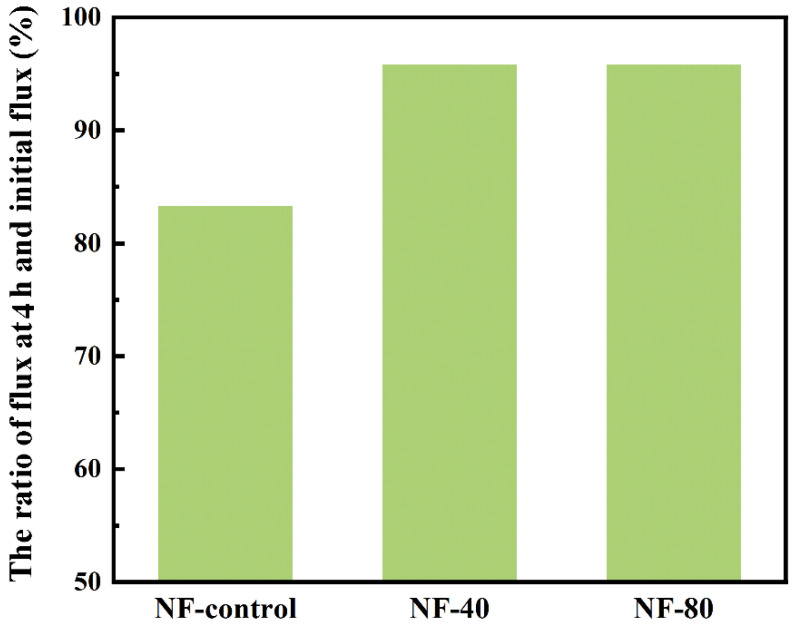
The ratio of flux at 4 h and initial flux, containing NF-control, NF-40, and NF-80 membranes after 4 h fouling test using 200 mg/L sodium alginate.

Figure 10 shows the changes of membrane flux for the NF-control, NF-40, and NF-80 with feeding solution containing 200 mg/L SA at the same initial flux after 4 h fouling test. The NF-40 and NF-80 membranes exhibited only slight water permeability decline of 4.2%, while the NF-control had a sharp decline of 16.7%. Typically, carboxyl groups on the selective layer provide potential bonding sites for foulants, thus accelerating the membrane fouling [43,44]. The complexation between Ca^2+^ and carboxyl groups enables the occupying of sites and effectively suppresses this fouling behavior. Additionally, the high hydrophilicity of the surface also contributes to the antifouling properties of NF-40 membrane [45]. 

Notably, the previous study showed that presence of Ca^2+^ in aqueous solution can accelerate the formation of gel network of foulants (e.g., SA) on membrane surface [46]. However, in this study, the intercalated Ca^2+^ performed antifouling performance which is surprisingly different from the role of Ca^2+^ in aqueous solution. It was ascribed that the intra-bridging of Ca^2+^ resulted in the shield of carboxyl groups and the increase of hydrophilicity, which reduced carboxyl-group-based foulant attachment on membrane surface. The intercalation of Ca^2+^ in PA matrix avoids its possible negative effects on membrane fouling (in the form of aqueous Ca^2+^).

## 4. Conclusions 

A “dynamic” modification based on Ca^2+^-carboxyl intra-bridging method was successfully introduced during post-treatment to improve the membrane performance. The membrane roughness significantly increased when calcium ions were added. XPS and ATR-FTIR characterization demonstrated that Ca^2+^ was chemically embedded in the PA layer. Surface hydrophilicity and charge were also changed, due to the strong complexation between Ca^2+^ and carboxyl groups and the embedding of Ca^2+^ in the PA layer. It is noted that the NF-40 membrane had excellent water permeability compared with NF-control, maintaining a high Na_2_SO_4_ rejection rate. Furthermore, the modified membranes showed antifouling performance. The changes of physicochemical properties are mainly associated with the formation of Ca^2+^-carboxyl chemical bonds and regulation of cross-linking process by the presence of Ca^2+^. This study highlights the importance of Ca^2+^-carboxyl intra-bridging post-treatment during the fabrication, which provides a simple and easy-to-operate way for fabricating high performance of TFC NF membranes. 

## Figures and Tables

**Table 1:** Surface element composition of the resulting membranes.

Sample	Element Content (%)			
	C	N	O	Ca
NF-control	76.86	8.51	13.81	0.45
NF-10	71.16	6.79	15.96	2.83
NF-20	67.74	5.16	15.3	4.52
NF-40	67.1	6.35	15.86	4.53
NF-80	69.14	5.53	14.65	4.04

**Table 2:** The performance comparison of NF-40 with other NF membranes with various post-treatment methods.

Membrane	Operating Pressure (bar)	Post-Treatment	Water Permeability (L·m^−2^·h^−1^·bar^−1^)	Na_2_SO_4_ Rejection (%)	References
TFC-M3	13.8	thermal treatment	13.6	97.7	[17]
PA@A-0	6	heat curing	16.7	97.4	[28]
PA-16	6	organic solution	7.6	94.9	[25]
PEI/TMC	4	ethanol	9.5	56.0	[38]
MWCNT-OH	6	heat-treatment	6.9	97.6	[39]
NFM-5	6	heat-treatment	15.2	97.0	[40]
NF-90	5	alkali solution	15.8	-	[41]
TFNC-2	13	hot pressing	22.3	92.0	[42]
NF-40	8	adding Ca^2+^	29.76	98.1	This work
